# The League of Mercy

**Published:** 1904-03-12

**Authors:** 


### THE LEAGUE OF MERCY.

It is interesting to note the position assumed by men and women in the metropolitan constituencies towards the question of personal service for the sick in the days of health. With the exception of Fulham, where both men and women have come forward in numbers in friendly competition as vice-presidents and members of the League of Mercy, most of the districts show that women are much more willing to render personal service than men in these days. In the Ealing division, of which Mr. Leopold de Rothschild is the President, his well-known interest in charity of all kinds has attracted a large number of men, with the result that there are 27 vice-presidents who are men, and only four lady vice-presidents. In South Paddington, on the other hand, it has been found practically impossible to induce the men at present to join the League of Mercy in any numbers. Seeing that at Fulham the friendly rivalry referred to has produced the maximum result in cash, we venture to hope that members of the medical profession and men of every degree will bestir themselves and lend a hand to the cause of the hospitals by becoming vice-presidents and members of the League of Mercy. The honorary secretaries of the League, 29 Southampton Street, Strand, will be glad to receive the name and address of anybody willing to give a little time to the work.

